# Acute kidney injury in cardiorenal syndrome type 1: a meta-analysis

**DOI:** 10.1186/cc13554

**Published:** 2014-03-17

**Authors:** W Vandenberghe, S Gevaert, H Peperstraete, I Herck, J Decruyenaere, E Hoste

**Affiliations:** 1 University Hospital Ghent, Belgium

## Introduction

Cardiorenal syndrome type 1 (CRS-1) reflects an abrupt worsening in cardiac function leading to acute kidney injury (AKI). Acute cardiac conditions contributing to CRS-1 include acute heart failure (AHF), acute coronary syndrome (ACS) and cardiac surgery (CS). The objective of this study was to evaluate the epidemiology of AKI in CRS-1.

## Methods

This is a systematic review and meta-analysis. AKI defined by the RIFLE definition and its modifications AKIN and KDIGO is grouped as AKIRIFLE. Similarly, AKI defined by variations of worsening renal failure is grouped as AKIWRF. Incidence of AKI is reported by the different definitions of AKI. In addition, we report on mortality and length of intensive care and hospital stay (LOSICU and LOShosp) for AKIRIFLE. Data are reported as percentage, risk ratio (RR), and mean difference (MD).

## Results

Our literature search yielded 316 potential papers, of which 57 were included (20 papers on AHF, 15 ACS and 22 Cs). A risk of bias analysis showed a low risk for selection bias in 55% of the studies and prospective data collection in 45%. AKIRIFLE was used in 33 studies (RIFLE in 22, AKIN in 14, KDIGO in four), AKIWRF, with six variants, in 24 studies and use of RRT (AKIRRT) in 20 studies. The incidence of AKI in CRS-1 patients defined by AKIRIFLE and AKIWRF was similar (22.5%, respectively 22.4%, *P *= 0.401), and greater than AKIRRT (2.6%, both *P *< 0.001). AKIRIFLE occurred more frequently in AHF patients compared with ACS and CS patients (55.0% vs. 14.9% vs. 19.3%; *P *= 0.009 respectively *P *= 0.001, *P *= NS for ACS vs. CS). This was similar when defined by AKIWRF. AKIRRT was evenly distributed among CRS- 1 subtypes (AHF 4.3%, ACS, 1.7%, and CS 3.1%, *P *= 0.611). Despite predominant low severity of AKIRIFLE (stage 1: 16.9%, stage 2: 3.7%, and stage 3: 3.6%), AKIRIFLE was associated with increased mortality (RR = 5.4), LOSICU (MD 1.7 days), and LOShosp (MD 4.4 days), and increasing AKIRIFLE severity was associated with increase in these three outcomes in all CRS-1 patients as well as in the three subgroups. The impact of AKIRIFLE on mortality was greatest in CS patients (AHF RR = 2.8, ACS RR = 3.5, and CS RR = 9.1). Not surprisingly, AkIWRF had similar impact on outcomes, but AKIRRT had greater impact compared with AKIRIFLE (mortality RR = 9.16, LOSICU MD = 10.6 days, and LOShosp, MD = 20.2 days).

## Conclusion

Almost one-quarter of patients with an acute cardiac condition had AKI, and RRT was used in approximately 3%. AKI was associated with significant worse outcomes. AHF patients experienced the highest incidence of AKI, but the impact on mortality was greatest in CS patients.

